# Germ cell development in the Honeybee (*Apis mellifera*); *Vasa *and *Nanos *expression

**DOI:** 10.1186/1471-213X-6-6

**Published:** 2006-02-17

**Authors:** Peter K Dearden

**Affiliations:** 1 Laboratory for Evolution and Development, Biochemistry Department, University of Otago, P.O. Box 56, Dunedin, New Zealand

## Abstract

**Background:**

Studies of specification of germ-cells in insect embryos has indicated that in many taxa the germ cells form early in development, and their formation is associated with pole plasm, germ plasm or an organelle called the oosome. None of these morphological features associated with germ cell formation have been identified in the Honeybee *Apis mellifera*. In this study I report the cloning and expression analysis of Honeybee homologues of *vasa *and *nanos*, germ cell markers in insects and other animals.

**Results:**

*Apis vasa *and *nanos *RNAs are present in early honeybee embryos, but the RNAs clear rapidly, without any cells expressing these germ cell markers past stage 2. These genes are then only expressed in a line of cells in the abdomen from stage 9 onwards. These cells are the developing germ cells that are moved dorsally by dorsal closure and are placed in the genital ridge.

**Conclusion:**

This study of the expression of germ cell markers in the honeybee implies that in this species either germ cells are formed by an inductive event, late in embryogenesis, or they are formed early in development in the absence of *vasa *and *nanos *expression. This contrasts with germ cell development in other members of the Hymenoptera, Diptera and Lepidoptera.

## Background

The formation and placement of primordial germ cells (PGCs) in animal embryos is of interest in developmental biology, evolutionary biology and biotechnology. In Diptera, including the well studied *Drosophila melanogaster*, PGCs form as morphologically and molecularly distinct pole cells, in the posterior of the embryo, early in development [1,2]. In insects outside the Diptera PGCs can develop in regions of pole or germ plasm, a distinct form of cytoplasm in early eggs [3], or are associated with the oosome [4-6] a specialised subcellular structure that appears to define PGC fate early in embryogenesis. These specialised structures and/or cytoplasm are widely distributed amongst insects but are not found in all insect embryos [1,7].

In the honeybee (*Apis mellifera*), little is known about the formation of PGCs in the embryo. In late embryonic and larval stages PGCs have been identified in the genital ridge situated near the dorsal surface of the abdomen where the gonads develop [8]. Previous authors have stated that there is no evidence for PGCs arising early in development [8] as is seen in other insects.

In other insects PGC placement and development has been studied using molecular markers of germ cell fate [7,9,10]. Two genes, *vasa *and *nanos*, have proved useful markers for PGCs in a broad range of species.

The *vasa *gene encodes a DEAD box RNA helicase that is expressed in the PGCs of all major groups of animals examined [11-17]. In mice, *vasa *related genes are expressed in PGCs and are required for PGC development [18]. In *Drosophila vasa *is required for multiple processes in the development and maintenance of PGCs (reviewed in [19]) including the localisation of *nanos *RNA [20].

*Nanos *genes encode zinc finger transcription factors that have been shown to be expressed in the PGCs of Diptera [21-23], *Caenorhabditis elegans *[24], Cnidarians [25,26], Leech [27,28], mice [29] and humans [30]. In *Drosophila nanos *is required for PGC migration and fate [21] and acts to repress somatic cell fate in PGCs by repressing differentiation [31,32]. In Zebrafish, *nanos *is required for PGC migration and survival [33]. Mice have three *nanos*-like genes, two of which are expressed in PGCs and required for their maintenance [29].

*Vasa *expression, assayed with in-situ hybridisation or antibodies, has been used most generally as a PGCs marker in arthropod embryos. Using both techniques, PGC specification has been studied in the orthopteran *Schistocerca gregaria*. In this species PGCs form at the dorsal boundaries of the germband in the abdomen and migrate dorsally to the gonads [7]. *Vasa *expression has also been examined in the lepidopteran *Bombyx mori *[9]. In this species, *vasa *RNA is first expressed in the presumptive embryo and then comes to be located in cells at the posterior of the germ band. In later stages these vasa positive cells populate the abdomen in regions consistent with the forming gonads. In non insect arthropods, *vasa *expression has also been examined in the two-spotted spider mite (*Tetranychus urticae*) [11], and the crustaceans *Daphnia *[12] and *Parhayle *[13]. In *Tetranychus*, *vasa *RNA marks a population of cells underlying the cellular blastoderm that migrate into the posterior of the embryo and are incorporated into the gonad. In *Daphnia*, vasa protein is localised in a subcellular compartment in 8 cell stage embryos and is partitioned into a single blastomere at the 16-cell stage. This vasa positive cell is the progenitor for the germ line in this species [12]. In *Parhayle*, vasa protein expression becomes apparent at the 8-cell stage in the g-micromere. Manipulation of the embryo, however, indicates that localised determinants for PGC development exist at the 2 cell stage [13].

In Hymenoptera, the insect order containing the honeybee, *vasa *expression has been examined only in the polyembryonic parasitic wasp *Copidosoma floridanum*. In this species, an organelle, the oosome, stains for *vasa *RNA and is segregated into cells that then express *vasa *RNA and protein and form PGCs. Inheritance of PGCs appears to regulate both larval caste and axis formation [10,34]. Oosomes that specify germ-cell fate are found in many hymenopteran species [4,6] but are not present in Honeybee ovaries or embryos [35].

*Nanos *genes have a role in specifying posterior regions of insect embryos [22,23,36-38]. In *Drosophila*, *nanos *RNA is localised to posterior regions of the embryo, and nanos translation is repressed in the rest of the embryo by the binding of smaug protein to a RNA secondary structure in the 3'UTR of the *nanos *mRNA [39]. Nanos protein then regulates Hunchback (Hb) translation by recruiting a cofactor, pumilio, that binds a 'nanos response element' in the 3'UTR of the Hunchback mRNA, thus restricting Hb protein expression to the anterior of the embryo [37]. This translation repression activity in the posterior of insect embryos appears to be conserved in the orthopteran *Schistocerca americana *[36] where *nanos *RNA is also posteriorly localised. Posteriorly localised *nanos *expression is also found in mosquito embryos [22].

In this paper I have cloned cDNA fragments of Honeybee *vasa *and *nanos *and used in-situ hybridisation on embryos and ovaries to determine if honeybee PGCs are specified early in development, like many holometabolous insect embryos, or if they form late in development from an inductive event.

## Results

### Identification of homologues of *nanos *and *vasa *in honeybees

Reciprocal blast searches [40] of the honeybee and *Drosophila melanogaster *genomes with *Drosophila vasa *and *nanos *sequences identified a single region of the honeybee genome with homology to *vasa*, and a single region with homology to *nanos*. Gene predictions corresponding to each of these regions were collected and analysed.

A predicted transcript (GB14804-PA, DQ288391) located on group 1.64 has high homology to *Drosophila vasa*. I term this transcript *Amvasa*. The transcript is predicted to encode a protein containing all of the 8 domains conserved in DEAD box helicases. The GIVGXA motif, conserved in insect vasas, is also conserved but the EXRKF domain conserved among Vasa and PL10 proteins is not present. The function of this motif is not known. The full length of this transcript was cloned from cDNA.

**Figure 1:**
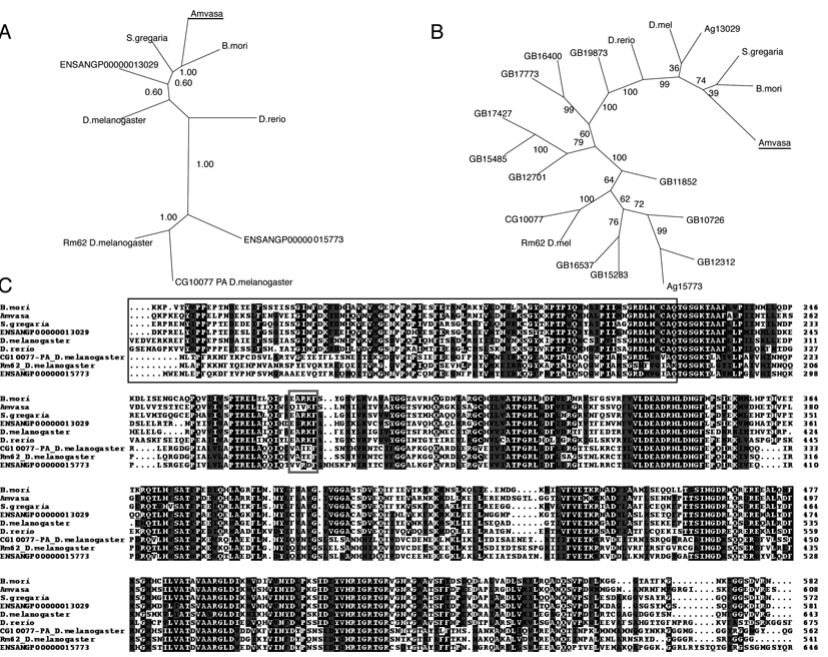
**Analysis of Apis *vasa***. A) Bayesian phylogeny of vasa protein sequences and Rm62-like DEAD box helicases from insects (and *Danio*). The predicted *Apis mellifera *vasa protein sequence clusters with other insect vasa proteins to the exclusion of Rm62 DEAD box helicases. ENSANGP00000013029 and ENGSANG00000015773 are *Anopheles *predicted protein sequences. This phylogram was derived from a multiple alignment shown in C. B) Neighbour joining phylogeny of vasa protein sequences and honeybee predicted DEAD-box containing proteins. *Amvasa *is the only *Apis *DEAD-box containing gene to cluster within the vasa clade. C) Multiple alignment of the most conserved domains of Vasa predicted proteins. The region boxed in red is the EXRKF motif that is not conserved in Honeybee vasa. The region boxed in black is the predicted protein encoded by the 3' end of the probe used for in-situ hybridisation.

Phylogenetic analysis of the *Amvasa *predicted protein sequence indicates that it clusters with *Drosophila*, *Bombyx *and *Schistocerca *vasa proteins as well as a predicted vasa-like dead box helicase from *Anopheles *(ENSANGP00000013029) (Figure 1A). Related non-vasa DEAD-box helicases form a separate clade in this analysis, indicating that the *Amvasa *is a homologue of *Drosophila vasa *and not a related DEAD-box helicase. The lack of the vasa specific EXRKF motif is unusual for an insect vasa protein. To ensure that the gene identified is the orthologue of vasa from the Honeybee, the Honeybee genome was searched for regions encoding DEAD box helicase proteins. All of the DEAD-box encoding regions have been identified in the GLEAN3 official gene prediction set. Neighbour joining phylogenetic analysis of a multiple alignment of the predicted proteins encoded by these sequences and other insect and vertebrate DEAD-box helicase proteins indicates that the *Amvasa *gene is the closest homologue of *vasa *in the Honeybee genome (Figure 1B)

**Figure 2:**
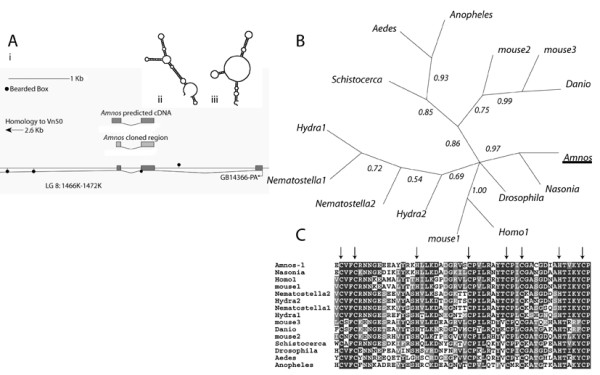
**Analysis of *Apis nanos***. A) i) Map of the genomic region that contains the highest similarity to nanos sequences in the bee genome. The map indicates the regions of similarity to *nanos *genes, the cDNA region cloned and the position of putative bearded boxes associated with *nanos *translation regulation in *Drosophila*. ii and iii) RNA structures of the first 130 bases of the *Drosophila nanos *(ii) and *Amnos *(iii) 3'UTRs predicted by MFOLD [42]. B) Bayesian phylogeny of nanos protein sequences derived from the alignment shown in D. The predicted *Apis mellifera *nanos protein sequence (Amnos, underlined) clusters with *Drosophila *and *Nasonia *nanos sequences with high posterior probability. C) Alignment of the most conserved regions of nanos protein sequences from metazoa.

The sequence with highest homology to *Drosophila nanos *in the Honeybee genome is included in a predicted transcript (GB14366-PA) that is annotated as similar to venom protein Vn50 (Located on group 8.7). Analysis of this transcript indicates that homology to *nanos *is restricted to two upstream exons almost 7 KB from the exons of the predicted transcript with homology to venom protein Vn50 (Figure 2A). Just downstream of the second exon corresponding to *nanos *is a gap in the genome sequence that may have caused an error with the gene prediction software. I have sequenced across this gap and identified an in-frame stop codon downstream of the second exon. Primers designed to amplify full-length *nanos *from cDNA indicate that the gene prediction is correct in this area. I designate the gene from which this sequence derives *Amnos*. This sequence is deposited in Genbank as DQ288392.

Multiple alignments of predicted proteins from nanos relatives indicate that the highest similarity between these sequences lies in the C-terminal zinc-finger DNA binding domain (Figure 2B). This region contains the two CCHC motifs for coordinating the zinc atoms that are completely conserved in *Amnos*. Outside of this region, sequence similarity is limited to short sequences. Phylogenetic analysis of a multiple alignment of this conserved DNA binding domain (Figure 2C) clusters the predicted Amnos protein sequence with a predicted nanos gene from *Nasonia*, and *Drosophila *nanos. Other insect nanos sequences (*Schistocerca*, *Aedes *and *Anopheles*, form a separate clade with mouse nanos 2 and 3 and *Danio *nanos. Mouse nanos1 and human nanos 1 sequences cluster with the *Drosophila *sequence and cnidarian nanos sequences form a separate cluster.

In *Drosophila *the 3'UTR of *nanos *contains a structural RNA motif required for translational repression [39,41], no sequence homology to this region can be seen in the *Amnos *3'UTR. An RNA structure prediction program (MFOLD[42]) predicts the first 130 bases of the *Amnos *3'UTR produces a similar RNA fold to that of the *Drosophila *3'UTR (Figure 2A ii and iii). The *Drosophila *motif contains sequences similar to bearded boxes that are also associated with translation repression [43]. Predicted bearded boxes are present downstream of the *Amnos *gene but not in the predicted secondary structure.

### Expression of *Amvasa *in honeybee embryos

**Figure 3:**
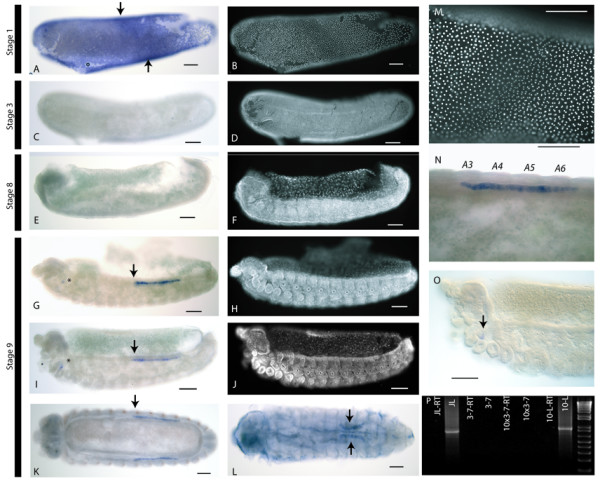
**Expression of *Amvasa *in *Apis mellifera *embryos**. Scale bars indicate 100 micrometers. A, C, E, G, and K, Brightfield images of embryos hybridised with probes against *Amvasa*, Lateral view. B, D, F, H, J, Fluorescent micrographs of the same embryos stained using the nuclear stain DAPI.A and B)) A stage 1 embryo stained (blue) for *Amvasa *RNA. Expression is present in the cytoplasm of all energids. The dark band running across the embryo (arrows) is due to mitosis occurring in that region (see B and magnified in M). C-F) embryos of stage 3 (C and D) and stage 8 (E and F) showing no expression of *Amvasa *RNA. G-J) embryos of stage 9 stained for *Amvasa *RNA. Expression can be seen in a line of cells starting in the 3^rd ^abdominal segment (arrow in G and I) and stretching back to the 6^th ^abdominal segment. The line of cells expressing *Amvasa *(magnified in N) lies under the epidermis and is just ventral to the boundary between the germband and the extra-embryonic membranes. A small group of cells expressing *Amvasa *can also be seen in the mandibular segment (asterisks in G and I). K) View of the dorsal surface of a stage 9 embryo stained for both *Amvasa *RNA (blue) and engrailed-like proteins (brown). Arrow marks the third abdominal segment. L) View of the dorsal surface of a just-hatched honeybee larva stained for *Amvasa *RNA. Expression can be seen in two crescents of cells near the midline of the larva (arrows) in the approximate position of the future gonads. The background in this larva is due to the forming cuticle. M) Magnification of the stage 1 embryo shown in A and B stained with the nuclear dye DAPI showing a region of mitoses in the embryo. N) Magnification of the *Amvasa *expressing cells of a stage 9 embryo, dorsal view, segments marked. O) Magnification of the anterior region of a stage 9 embryo stained for *Amvasa *RNA. A group of cells in the mandibular segment are weakly expressing *Amvasa *RNA (arrow). P) RT-PCR analysis of *Amvasa *expression. RT-PCR was carried out on RNA from just-laid eggs (JL), eggs between stages 3 and 7 (3–7) and eggs older than stage 10 (10-L). Each RT-PCR experimental lane lies next to a control reaction where amplification was from RNA treated as for the experimental lanes but without the addition of reverse transcriptase in the first stand synthesis. This controls for genomic DNA contamination of the cDNA. These lanes are labelled with -RT. RT-PCR was also attempted from ten times more cDNA from stages 3–7 in an attempt to detect low level expression (10 × 3–7) Successful amplification of *Amvasa *gives a 916 bp fragment. Amplification is seen only in cDNA from just laid embryos and stages 10 and above.

In-situ hybridisation using a fragment of the *Amvasa *cDNA sequence was used to determine the placement and development of PGCs in female honeybee embryo (Figure 3). The probe was made from a 631 bp fragment of *Amvasa *from the second exon, a relatively non-conserved region of the gene. The C terminal part of the protein encoded by this fragment is boxed in Figure 1. *Amvasa *RNA is present at early stages of *Apis *embryogenesis, with a uniform distribution at stage 1 (Staging as per [35]) (Figure 3A and 3B). This initial expression rapidly disappears by late stage 1. *Amvasa *in not expressed in any cells until stage 9, when *Amvasa *RNA appears in a line of cells starting in the 3^rd ^abdominal segment (A3) and stretching to the 6^th ^abdominal segment (A6) (Figure 3G and 3N). This line of cells lies just ventral to the boundary between the germband and the extra-embryonic membranes and lies just underneath the epidermis of the embryo. These cells continue to express *Amvasa *through the rest of embryogenesis (Figure 3I and 3K), and, in larval honeybees, come to be placed in regions consistent with the placement of the genital ridge (Figure 3L).

*Amvasa *RNA is also detected in a small group of cells in the mandibular segment in stage 9 embryos (Asterisks in figure 3G and 3I and arrow in Figure 3O). It is unclear what this structure is.

The expression of *Amvasa *RNA seems to indicate that PGCs in *Apis *form late in embryogenesis. It is possible, however, that the distribution of the *Amvasa *RNA does not reflect the distribution of *Amvasa *protein, as in *Drosophila *[44]. To determine if *Amvasa *protein is present in embryos stages 2–8, embryos were stained using an antibody raised against *Schistocerca gregaria *vasa that has been shown to cross react in a number of species [7,13,25]. No staining above background in *Apis *embryos or ovaries was detected using the antibody in conditions in which *Drosophila melanogaster *embryos did stain in the canonical vasa pattern, implying this antibody does not cross-react with *Amvasa *(data not shown).

To confirm the timing of *Amvasa *RNA expression, RT-PCR was carried out on RNA from honeybee embryos of various stages (Figure 3P). A PCR product of the target length, consistent with *Amvasa *RNA, was found only in RNA from just laid eggs and embryos over stage 10. No *Amvasa *RNA could be detected in embryos of stages 3–7, consistent with our in-situ hybridisation results.

### Expression of *Amnos *in honeybee embryos

**Figure 4:**
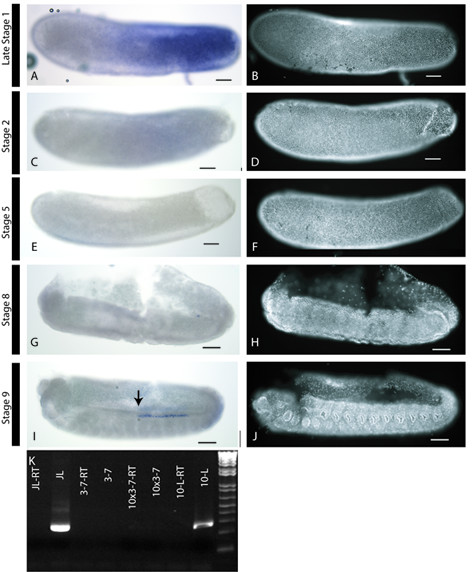
**Expression of *Amnos *in *Apis mellifera *embryos**. Scale bars denote 100 micrometers. A, C, E, G and I, Brightfield images of embryos hybridised with probes against *Amnos*, Lateral view. B, D, F, H, J, Fluorescent micrographs of the same embryos stained using the nuclear stain DAPI.A and B) A late stage 1 embryo stained for *Amnos *RNA. *Amnos *RNA is present in a gradient with highest concentrations at the posterior pole. C and D) a stage 2 embryo stained for *Amnos *RNA. Faint staining is present in a similar pattern to that in A. *Amnos *RNA expression fades very fast in stage 2 embryos. E-H) Stage 5 (E and F) and stage 8 (G and H) embryos stained for *Amnos*. No expression is seen. I and J) Stage 9 embryo stained for *Amnos *RNA, expression is seen in a line of cells starting in the third abdominal segment (arrow in I) and stretching to the 6^th ^abdominal segment. The *Amnos *expressing cells underlie the ectoderm of the embryo and lie just ventral to the border between the germband and the extraembryonic membranes. K) RT-PCR analysis of *Amnos *expression. RT-PCR was carried out on RNA from just-laid eggs (JL), eggs between stages 3 and 7 (3–7) and eggs older than stage 10 (10-L). Each RT-PCR experimental lane lies next to a control reaction where amplification was from RNA treated as for the experimental lanes but without the addition of reverse transcriptase in the first stand synthesis. This controls for genomic DNA contamination of the cDNA. These lanes are labelled with -RT. RT-PCR was also attempted from ten times more cDNA from stages 3–7 in an attempt to detect low level expression (10 × 3–7) Successful amplification of *Amnos *gives a 257 bp fragment. Amplification is seen in cDNA from just laid embryos (JL), and cDNA stages 10 and above.

As *Amvasa *RNA expression indicated the late formation of PGCs and I was unable to confirm this using the anti-vasa antibody, the expression of *Amnos *was used as a second PGC marker in female embryos. *Amnos *RNA is first detected in stage 1 embryos where it is present in a gradient with highest expression in posterior regions of the embryo (Figure 4A). This expression fades rapidly through embryogenesis such that it is faint in stage 2 embryos (Figure 4C) and absent from stage 3 embryos. No further expression of *Amnos *is seen in embryos until stage 9 (Figure 4I), when expression appears in a line of cells, starting in the 3^rd ^abdominal segment and stretching to the 6^th ^abdominal segment. Like the cells marked with *Amvasa*, *Amnos *expressing cells lie under the epidermis and just ventral to the boundary between the embryonic and extraembryonic membranes. The distribution of *Amnos *and *Amvasa *in these embryos is identical except for the anterior patch of cells expressing *Amvasa*; these cells do not express *Amnos*.

To confirm the timing of *Amnos *RNA expression, RT-PCR was carried out on RNA from honeybee embryos of various stages (Figure 4K). A PCR product of the target length (257 bp), consistent with *Amnos *RNA, was found only in RNA from just-laid eggs and embryos over stage 10. These data are consistent with our in-situ hybridisation results in that *Amnos *RNA is not expressed between stages 3–7.

### Expression of *Amvasa *in queen and worker bee ovaries

**Figure 5:**
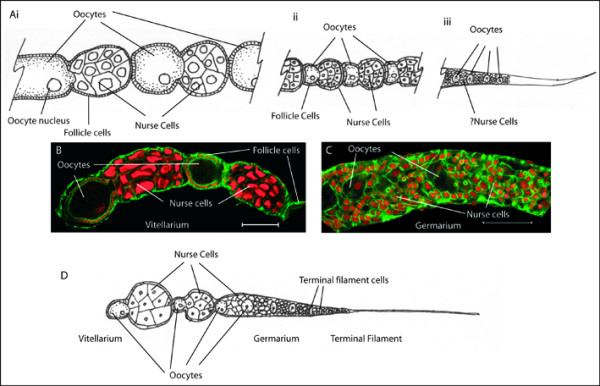
**Structure of the Honeybee ovary**. A) Diagram of the morphology of an ovariole from a mated queen bee ovary. i) the late vitellarium, ii) the early vitellarium, iii) the germarium and terminal filament (the placement of germ cells in the terminal filament is unclear and thus not diagrammed). B) A projection of 25 confocal Z sections through the late germarium of a mated honeybee queen ovariole stained for DNA using Propidium iodide (red) and cortical actin using Alexa fluor 488 phalloidin (green). C) A single confocal section through the germarium of a honeybee queen ovariole, stained as per B, Note the circular actin-rich structures, possibly ring canals. D) Diagram of the morphology of a worker bee ovariole.

**Figure 6:**
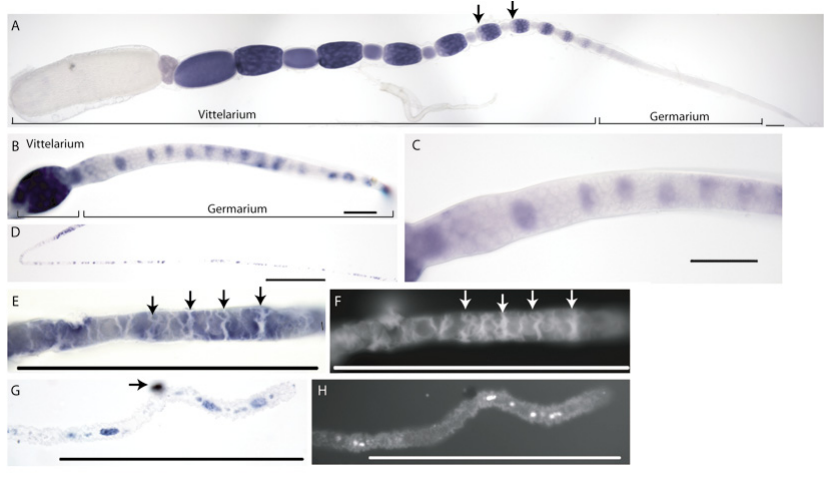
**Expression of *Amvasa *in Honeybee ovaries**. Scale bars represent 100 micrometers. A) The full length of a mated queen ovariole stained for *Amvasa *RNA. *Amvasa *RNA is present in both nurse cells and oocytes but not follicle cells. Staining is absent from the oldest oocytes, an artefact caused by problems getting labelled probes into these oocytes. All other oocytes have uniform staining of *Amvasa *RNA except those in the germarium and terminal filament where *Amvasa *RNA is absent. *Amvasa *RNA is also present in the nurse cells. In the early vitellarium, the nurse cells closest to the oocyte express lower levels of *Amvasa *RNA (arrows), but this difference in expression is not seen in the late vitellarium. *Amvasa *RNA levels are reduced in the last set of nurse cells, which are degrading prior to fertilisation and oviposition of the egg. B) *Amvasa *RNA expression in the vitellarium and germarium of a worker bee ovary. In the vitellarium, *Amvasa *RNA is expressed in the nurse cells and oocyte. In the germarium, *Amvasa *RNA is restricted to the oocytes themselves, except for the last oocyte/ nurse cell group, where *Amvasa *is expressed in some of the nurse cells. C) Magnification of the germarium shown in B under DIC optics showing *Amvasa *expression in germ-cells and in the last group of nurse cells (arrowed) D) The tip of the germarium and the terminal filament of a worker bee ovary stained for *Amvasa *RNA. Amvasa expression appears in a subset of cells in each tissue, presumably PGCs. E and F) Magnification of the beginning of the terminal filaments of a worker bee ovary stained for *Amvasa *RNA and using the nuclear dye DAPI under brightfield (E) and fluorescence (F) optics. *Amvasa *RNA is seen in a subset of cells in this region. The terminal filament cells (arrowed) have no *Amvasa *expression. G and H) a region of terminal filament from a worker bee ovary stained for Amvasa, and using the nuclear dye DAPI under brightfield (G) and fluorescence (H) optics. Cells expressing *Amvasa *can be seen surrounded by non-staining cells. *Amvasa *expression quenches the nuclear signal in these images, so bright spots of DAPI fluorescence indicate unstained nuclei.

*Apis mellifera *have polytrophic meroistic ovaries, similar to those of *Drosophila melanogaster*. Despite this similarity, a number of differences in morphology and biology exist. One particularly important difference is that worker bee ovaries have many less ovarioles than those of queens (Figure 5). Workers in a queenright colony have small ovaries that are chemically repressed by the presence of the queen and her eggs [45]. Removal of the queen from a colony can cause the reactivation of the worker bee ovaries and the workers may lay eggs [45]. To see if germ cell placement or organisation is significantly different in workers compared to queen ovaries I used in-situ hybridisation for *Amvasa *RNA to examine the placement of germ cells in both types of ovary (Figure 6).

In the queen ovary, both nurse and germ cells express *Amvasa*. *Amvasa *RNA is not localised to any particular area in either cell type. *Amvasa *RNA is present in oocytes in the vitellarium, but expression is absent, or very weak in the germarium and terminal filament. In the nurse cells, RNA is present in all cells in the vitellarium, but in the germarium, the expression of *Amvasa *RNA is reduced in the nurse cells closest to the oocytes (arrows in figure 6A). Expression of *Amvasa *RNA does not extend beyond the germarium in nurse cells. No expression of *Amvasa *is seen in follicle cells.

In worker ovaries a similar expression pattern can be seen (Figure 6B). *Amvasa *RNA is present in all oocytes, but, in contrast to queen ovaries, this expression extends into the terminal filament (Figures 6D, E, F, G, and 6H). Expression of *Amvasa *RNA in the nurse cells is reduced in worker ovaries. Expression can be seen in nurse cells in the vitellarium, and, faintly, in the nurse cells associated with the last oocyte in the germarium (Figure 6C). In the terminal filament (Figures 6D, E, F, G and 6H) *Amvasa *RNA expression marks germ cells in the entire structure. Comparison of the expression of *Amvasa *RNA and the location of nuclei stained with DAPI indicates that the germ cells in the start of the terminal filament are surrounded by disc shaped 'terminal filament cells' [46] that do not stain for *Amvasa*. However within these compartments, cells that do not express *Amvasa *are also present (Figure 6E and 6F). It is not clear if these are nurse cells or follicle cells. In the last sections of the terminal filament, cells positive for *Amvasa *RNA are interspersed with cells that are not (Figure 6G and 6H). The expression of *Amvasa *in the worker, but not queen terminal filament is a clear difference between castes.

### Expression of *Amnos *in queen and worker bee ovaries

**Figure 7:**
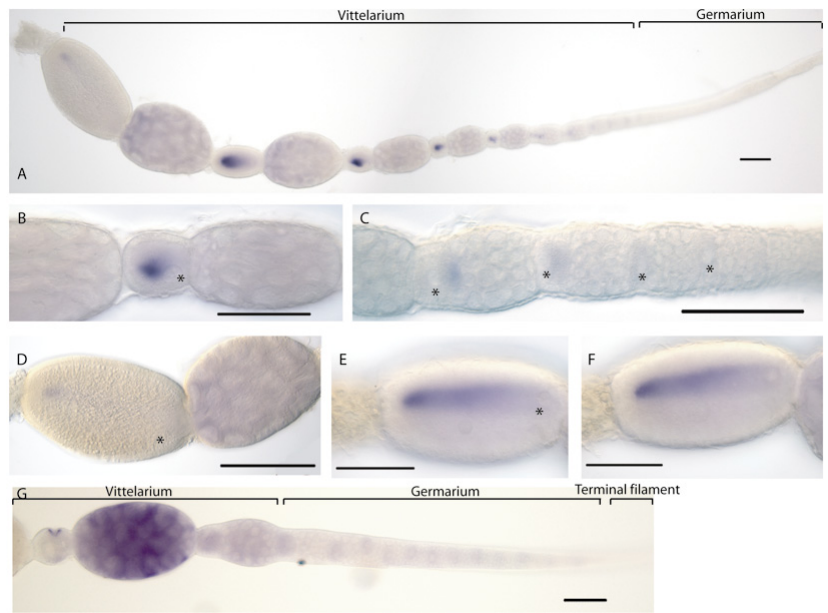
**Expression of *Amnos *in honeybee ovaries**. Scale bars represent 100 micrometers. A) Ovariole from a mated queen bee stained for *Amnos *RNA. *Amnos *RNA is present in both oocytes and a subset of nurse cells. In oocytes in the late vitellarium, *Amnos *RNA is present in a localised domain in the posterior regions of the oocyte, with a 'tail' of *Amnos *RNA spreading back along one side of the oocyte towards the anterior. In the early vitellarium, *Amnos *RNA is localised in a domain in central regions of each oocyte. *Amnos *is also expressed weakly in the nurse cells, particularly in those closest to the oocyte. B) DIC image of an oocyte and nurse cells from the early vitellarium showing the localised domain of *Amnos *and the nucleus (asterisk). The patch of *Amnos *RNA is not associated with the nucleus. C) DIC image of the basal germarium of an ovariole stained for *Amnos *RNA. In the germarium, oocytes contain *Amnos *RNA. In late germarium, this is in a patch as seen in the vitellarium. In earlier oocytes, expression appears uniform. The patch of *Amnos *RNA is not associated with the oocyte nucleus (asterisks). No expression of *Amnos *is seen in the nurse cells. D) DIC image of an oocyte and nurse cells from the late vitellarium. *Amnos *RNA is only present in a small posterior patch, not associated with the nucleus (asterisk). The nurse cells all express *Amnos *weakly. E and F) DIC images of two focal planes through an oocyte in the vitellarium stained for *Amnos *RNA. *Amnos *RNA is present in a streak running down the oocyte, with the highest and most defined expression in the posterior. The streak and domain is not associated with the oocyte nucleus (asterisk in E). G) Worker bee ovariole stained for *Amnos *RNA. Strong expression is seen in the nurse cells of the vitellarium, with weaker expression in the oocyte. In the germarium, *Amnos *RNA is weakly expressed in oocytes only. No expression is present in the terminal filament (data not shown).

The expression if *Amnos *RNA in queen and worker bee ovaries was also examined using in-situ hybridisation (Figure 7). In the early vitellarium and germarium, *Amnos *RNA is present only in a discrete domain in the anterior region of each oocyte, anterior to the oocyte nucleus (Asterisks in figure 7C). In later vitellaria, *Amnos *RNA staining is found faintly in the nurse cells, and in the localised domain in each oocyte. In later vitellaria, however, this domain lies on one side of the oocyte and appears as a patch in posterior regions of the egg with a more diffuse 'tail' stretching back to the more posterior regions. Neither the patch nor the tail are associated with the oocyte nucleus (asterisks in Figure 7B, C, D and 7E) and in all cases lies posterior to it. In the oldest oocytes (Figure 7D) *Amnos *RNA is found in an anterior patch that is fainter that that in younger oocytes. I believe this faint staining is an artefact caused by differential permeability of old and young oocytes. The 'comet-like' form of the patch of localised Amnos RNA in later vitellaria is consistent with movement of a discrete focus of Amnos RNA from anterior of the oocyte nucleus to posterior regions of the oocyte.

In worker bee ovaries, by contrast, *Amnos *RNA is weakly and uniformly distributed in oocytes in the vitellarium and germarium (Figure 7G[47]) and, at higher levels, in the nurse cells of the oldest oocyte. *Amnos *RNA is not expressed in nurse cells, follicle cells or cells in the terminal filament.

## Discussion

### Identification of *Apis *homologues of *nos *and *vasa*

Blast searches and phylogenetic reconstruction indicate that I have identified honeybee homologues of the evolutionarily conserved germ-cell markers *nanos *and *vasa*.

The *Amvasa *gene, also identified by [10], has all of the conserved sequences indicative of a vasa protein, and clusters with other insect vasa sequences to the exclusion of non vasa DEAD box helicases. The sequence does, however, have significant variation in the EXRKF motif, that is conserved in PL10 and vasa helicases. The function of this motif is unknown, thus the significance of its absence from *Amvasa *is not known.

The *Amnos *gene identified in this study is similar in C-terminal regions to nanos proteins from other species. This region contains the zinc coordination residues of the zinc fingers. These residues are completely conserved in *Amnos*. *Drosophila nanos *contains a sequence that forms a specific secondary structure in the 3'UTR that is required for translational repression [41]. This secondary structure is bound by the smaug protein in early embryos [39]. A similar secondary structure is predicted to form in the *Amnos *3'UTR though its function, if any, is unknown.

The conservation and phylogenetic placement of these sequences is consistent with them encoding the conserved germ-cell markers *vasa *and *nos*.

### Germ cell development in *Apis mellifera*

Expression of *Amnos *and *Amvasa *RNA occurs in two phases of honeybee embryonic development. Both genes are expressed early in development, *Amvasa *in the entire embryo, and *Amnos *in a gradient pattern with highest concentrations in the posterior of the embryo, both probably due to maternal RNA contribution. These expression patterns disappear by stage 2. In later development, at and after stage 9, both genes are expressed in a subset of cells that lie close to the dorsal boundary of the embryo proper and the extraembryonic membranes. These cells eventually come to be located in the dorsal-most regions of the embryo consistent with the genital ridges and forming gonads. That both genes are not expressed in embryos between stage 2 and stage 9 implies that during these stages there are no PGCs in the honeybee embryo, and that these cells form via an inductive event at, or just before, stage 9.

Neither morphological studies, nor the use of molecular markers has found any evidence for pole-cells, pole plasm or early segregating PGCs [8]. This is unlike the formation of PGCs in *Drosophila*, which has a similar long-germ form of embryo to that of *Apis*.

The expression of these two PGC markers implies that germ-cells form very close to the boundary of the embryo proper and the extra-embryonic membranes. The cells form in A3-A6 and no sign of migration of the PGCs can be seen. As dorsal closure occurs, the cells appear to be moved, passively, to the dorsal surface of the larva, close to the dorsal midline.

These findings indicate two possible scenarios for the origin of PGCs in the honeybee embryo. It is possible that the appearance of PGC markers at a late stage of embryogenesis, similar to the situation in mice [48,49] implies that an inductive event at stage 9 specifies PGCs. The organisation of the cells, in a single line, is what might be expected if the cells are being induced by a signal released from an almost linear source. Just dorsal of the PGCs are the extra-embryonic membranes that cover the dorsal opening. It is possible that the putative inducing signal leading to the formation of PGC fate emanates from the extra-embryonic membranes.

It is also possible that the *Amvasa *and *Amnos *RNA are not expressed in germ-cells that do form early in development, and only come to be expressed when these cells reach the abdomen. *Drosophila vasa *RNA expression is indeed absent from PGCs until stage 12 [44] and *nanos *expression is down-regulated in *Drosophila *after stage 10 [38]. In the crustacean *Parhayle *PGCs are specified very early in development, without the expression of *vasa *[13]

This study is unable to determine which of these two possible mechanisms underlies germ-cell formation in Honeybees.

### Localisation of nos RNA and posterior development in *Apis mellifera*

In *Drosophila *and other insects *nanos *acts to regulate posterior development [38]. *Nos *expression has been studied in both *Drosophila *[38,41], other dipterans [22,23] and *Schistocerca *[36]. In each studied species *nos *RNA is localised in posterior regions in early development. In *Drosophila *most *nos *RNA is localised very tightly to the posterior, and its translation is repressed in anterior regions [41]. In *Drosophila nos *acts as a key regulator of posterior development, translationally repressing, in concert with pumilio, the anterior gene *hunchback *in the posterior [37,50]. In *Schistocerca*, the expression of *nos *is coincident with a clearance of hunchback expression from the very posterior regions of the embryo [36]. *Hunchback *mRNA from *Schistocerca *is predicted to contain a nanos response element, similar to that which is bound by *Drosophila *nanos to repress hunchback translation.

In Honeybees *Amnos *RNA is present in the posterior of the early embryo (appearing in just laid eggs and clearing in stage 2) This domain is broader that that in *Drosophila *where the highest levels of *nos *RNA form, and appears to represent a concentration gradient, with highest RNA expression in the very posterior of the embryo. The *Amnos *3'UTR does not contain an element with sequence similarity to the stem-loop structure in the 3'UTR of *Drosophila nos*, but a similar RNA structure is predicted to be formed by it.

In the ovary of queen bees, *Amnos *RNA expression is found in a localised patch in oocytes, that appears to move, during oocyte maturation, from anterior to posterior. In *Schistocerca *[36], and mosquitoes [22]*nanos *RNA is also localised tightly to posterior regions of the oocyte. The localisation of *Amnos *RNA in the oocyte is consistent with a role in posterior patterning.

## Conclusion

I have used *Amvasa *and *Amnos *as molecular markers of PGC fate in the honeybee *Apis mellifera*. The timing of placement of their expression provides no evidence for early specification of germ-cells but implies that honeybee PGCs form from an inductive event late in embryonic development. It is also possible that germ-cells form early in the Honeybee embryo but do so in the absence of *vasa *or *nanos *expression. Both these possibilities differ from PGC specification in *Drosophila*, where morphologically distinct PGCs appear early in development and are marked by the expression of *vasa *and *nanos*, or the more closely related *Copidosoma*, where a cellular organelle determines PGC fate and contains vasa protein [5].

Any putative induction process implied by these results appears similar to the segregation of PGCs in *Schistocerca*, where early segregation is also not seen. The placement of PGCs, forming close to the border between extra-embryonic and embryonic tissue and inside the epidermal layer, seems similar in both species.

This mode of PGC formation indicates that the evolutionary history of PGC segregation in insects is not simple, with different mechanisms acting in different species. It is unclear, at this point, what the ancestral mechanism is, and how the different mechanism of PGC specification in extant insects may have arisen.

## Methods

### Beekeeping

*Apis mellifera *were cultured using standard techniques in Dunedin, New Zealand. Honeybee embryos were collected from frames removed from nucleus boxes containing small honeybee colonies.

### Gene identification and phylogenetics

Homologues of the *Drosophila melanogaster vasa *and *nanos *genes were identified in the Honeybee genome sequence (version 2) using tBlastN searches [40]. Regions of the genome with significant blast hits were extracted and blasted back to the *Drosophila melanogaster *genome. Gene predictions from either NCBI (using gnomon) or ENSEMBL were then examined in the regions with reciprocal top blast hits for *vasa *and *nanos*. Primers for amplification from cDNA were designed to the regions with the highest homology.

Multiple alignments of the predicted *Apis *genes with homologous genes from other species were carried out using ClustalX [51]. Phylogenetic analysis was performed on these multiple alignments using MrBayes 3.1 [52] or Phylip [47]

### Molecular Cloning

Ovaries were dissected from mated queen bees in PBS and poly A+ RNA extracted using a quickprep RNA extraction kit (GE Biosciences). cDNA was generated from this RNA using superscript II reverse transcriptase (Invitrogen) and an oligo-DT primer following the manufacturers instructions. PCR was performed on this cDNA using primers for vasa (tggcaatgtaacgataaaaagacc, AmvasaRNA5' tgggcgacacgatgacaac, AmvasaRNA3') or nanos (gtctccacacgcaccacaa, AmNanos5' acgccgcaagaaaaataagaaac, AmNanos3'). PCR products of the correct size were cloned into pGEM-T-Easy (Promega) following the manufacturers instructions. Plasmid DNA was isolated from these clones and sequenced at the Allan Wilson Centre for Molecular Ecology and Evolution.

RNA for RT-PCR experiments was extracted with an RNAeasy kit (Qiagen), treated with RNAse free DNAse (Invitrogen) for 2 hours at 37 degrees, heated to 65 degrees for 30 minutes, and precipitated with ammonium acetate and isopropanol. First strand synthesis and PCR were performed as above with primers for *Amvasa *(gcgtttccacccatcatc and gttgtcatcgtgtcgccca and *Amnos *(as above).

### In-situ hybridisation and antibody staining

In-situ hybridisation was performed on ovaries and embryos as described in Osborne and Dearden [53] and antibody staining after in-situ hybridisation using the 4D9 anti-engrailed-like antibody [54] as described in Osborne and Dearden [55].

### Confocal microscopy

Ovaries were dissected from mated queen bees and fixed in a mixture of 4% formaldehyde in PBS: Heptane overnight. Ovaries were washed in methanol and rehydrated in PTw (PBS + 0.1% Tween 20). The ovaries were treated with 1 μg/ml RNAse A and PTw for one hour, and then treated with 0.5 μg/ml Propidium iodide and 0.33 mM Alexa-Fluor 488 Phalloidin (Molecular probes) overnight in PTw. The ovaries were washed in PTw four times over 30 minutes and individual ovarioles dissected from the ovary mass. Individual ovarioles were mounted on microscope slides in 70% glycerol. Confocal imaging was carried out using a Leica confocal microscope.

## Authors' contributions

PKD conceived and carried out the experiments discussed above and drafted the manuscript.
